# Multiple Sclerosis: Modulation of Toll-Like Receptor (TLR) Expression by Interferon-β Includes Upregulation of TLR7 in Plasmacytoid Dendritic Cells

**DOI:** 10.1371/journal.pone.0070626

**Published:** 2013-08-12

**Authors:** Katja Derkow, Jakob M. J. Bauer, Michael Hecker, Brigitte K. Paap, Madhan Thamilarasan, Dirk Koczan, Eckart Schott, Katrin Deuschle, Judith Bellmann-Strobl, Friedemann Paul, Uwe K. Zettl, Klemens Ruprecht, Seija Lehnardt

**Affiliations:** 1 Department of Neurology, Charité - Universitätsmedizin Berlin, Berlin, Germany; 2 Department of Neurology, University of Rostock, Rostock, Germany; 3 Institute of Immunology, University of Rostock, Rostock, Germany; 4 Department of Hepatology and Gastroenterology, Charité - Universitätsmedizin Berlin, Berlin, Germany; 5 Clinical and Experimental Multiple Sclerosis Research Center, Charité - Universitätsmedizin Berlin, Berlin, Germany; 6 Cluster of Excellence NeuroCure, Charité - Universitätsmedizin Berlin, Berlin, Germany; 7 Experimental and Clinical Research Center, Charité - Universitätsmedizin Berlin and Max-Delbrück Center for Molecular Medicine, Berlin, Germany; 8 Institute of Cell Biology and Neurobiology, Center for Anatomy, Charité - Universitätsmedizin Berlin, Berlin, Germany; Centre d'Immunologie de Marseille-Luminy, CNRS-Inserm, France

## Abstract

Interferon-β is an established treatment for patients with multiple sclerosis (MS) but its mechanisms of action are not well understood. Viral infections are a known trigger of MS relapses. Toll-like receptors (TLRs) are key components of the innate immune system, which sense conserved structures of viruses and other pathogens. Effects of interferon-β on mRNA levels of all known human TLRs (TLR1-10) and the TLR adaptor molecule MyD88 were analyzed in peripheral blood mononuclear cells (PBMCs) of healthy donors by quantitative real-time PCR and by transcriptome analysis in PBMCs of 25 interferon-β-treated patients with relapsing-remitting MS. Regulation of TLR protein expression by interferon-β was investigated by flow cytometry of leukocyte subsets of healthy subjects and of untreated, interferon-β-, or glatiramer acetate-treated patients with MS. Interferon-β specifically upregulated mRNA expression of TLR3, TLR7, and MyD88 and downregulated TLR9 mRNA in PBMCs of healthy donors as well as in PBMCs of patients with MS. Plasmacytoid dendritic cells (pDCs) were identified as the major cell type responding to interferon-β with increased expression of TLR7 and MyD88 protein. In line with this, expression of TLR7 protein was increased in pDCs of interferon-β-treated, but not untreated or glatiramer acetate-treated patients with MS. Interferon-β-induced upregulation of TLR7 in pDCs is of functional relevance since pre-treatment of PBMCs with interferon-β resulted in a strongly increased production of interferon-α upon stimulation with the TLR7 agonist loxoribine. Flow cytometry confirmed pDCs as the cellular source of interferon-α production induced by activation of TLR7. Thus, upregulation of TLR7 in pDCs and a consequently increased activation of pDCs by TLR7 ligands represents a novel immunoregulatory mechanism of interferon-β. We hypothesize that this mechanism could contribute to a reduction of virus-triggered relapses in patients with MS.

## Introduction

Interferons were originally described as a family of antiviral proteins, owing to their capacity to interfere with viral replication [1]. Based on several pivotal clinical trials, interferon-β, a type I interferon, was approved as the first disease-modifying therapy for multiple sclerosis (MS), a chronic inflammatory CNS disease, in the 1990ies [2]–[4]. While interferon-β consistently reduces relapse rates by about one third and decreases disease activity as measured by magnetic resonance imaging in patients with relapsing-remitting MS (RRMS), the mechanisms of action underlying these effects are not well understood [5].

Toll-like receptors (TLRs) are pattern-recognition receptors, which recognize conserved structures of microbial pathogens, referred to as pathogen-associated molecular patterns [6], [7]. They are key components of the innate immune system whose activation orchestrates inflammatory responses and primes antigen-specific adaptive immunity. In humans, 10 functional TLRs (TLR1-10) have been identified so far. All TLRs are transmembrane proteins that can roughly be divided into cell surface-associated TLRs (TLR1, TLR2, TLR4-6) primarily sensing microbial membrane components, and TLRs located in intracellular vesicles (TLR3, TLR7-9) detecting viral or bacterial nucleic acids [6]. Except for TLR3, all TLRs recruit the Toll/interleukin-1 receptor (TIR) domain-containing adaptor molecule myeloid differentiation primary response gene 88 (MyD88) for activation of a complex intracellular signaling cascade [6].

Here, we hypothesized that one mechanism of action of interferon-β in MS may be related to regulation of TLRs. We thus performed a comprehensive study on the impact of interferon-β on mRNA expression levels of TLR1-10 and MyD88 in peripheral blood mononuclear cells (PBMCs) of healthy donors and of interferon-β-treated patients with RRMS. We observed an upregulation of TLR3, TLR7, and MyD88 and a downregulation of TLR9 by interferon-β in PBMCs of healthy donors as well as in PBMCs of patients with RRMS. Plasmacytoid dendritic cells (pDCs) were identified as the main cell type upregulating TLR7 and MyD88 protein in response to interferon-β and TLR7 was increased in pDCs of interferon-β-treated patients with MS. As a functional consequence, upregulation of TLR7 was accompanied by an enhanced production of interferon-α upon TLR7 stimulation. These results suggest that upregulation of the virus-detecting immune receptor TLR7 in pDCs and a subsequently enhanced activation of pDCs by TLR7 ligands may be a novel immunoregulatory mechanism of interferon-β in patients with MS.

## Materials and Methods

### Ethics statement

The study was approved by the institutional review boards of Charité - Universitätsmedizin Berlin (EA1/182/10) and Universität Rostock (II HV 27/2003). Written informed consent was obtained from all healthy donors and patients participating in the study.

### Cell separation, cell culture, and stimulation assays

Approximately 30 ml of peripheral venous blood were collected from patients treated at the Department of Neurology or NeuroCure Clinical Research Center, Charité - Universitätsmedizin Berlin, with a diagnosis of a clinically isolated syndrome (CIS) suggestive of MS or early RRMS (disease duration ≤2 years), according to the McDonald 2005 criteria [8]. Samples were processed within 1 hour and PBMCs were isolated by ficoll density gradient centrifugation. PBMCs were frozen immediately by using freezing medium containing 10% dimethyl sulfoxide and a standard freezing container with isopropyl alcohol. Subsequently, cells were thawed and used for flow cytometry analysis. PBMCs from healthy donors were generated likewise and used directly after isolation for *in vitro* stimulation assays. Freshly isolated PBMCs from healthy donors were cultured in RPMI 1640 medium supplemented with 10% FCS and 1% penicillin/streptomycin at a density of 2×10^6^ cells/ml in the absence or presence of 10, 100, or 1000 U/ml interferon-β-1b (Betaferon®, Bayer Pharma AG, Berlin, Germany) dissolved in NaCl solution (5,4 mg/ml). For RNA extraction cells were harvested after 6, 24, and 48 hours of treatment and for FACS analysis after 16 or 24 hours of treatment.

### RNA isolation and cDNA synthesis

Total RNA was extracted from PBMCs using the RNeasy Mini Kit (Qiagen, Hilden, Germany). Genomic DNA was eliminated by on-column DNase digestion (RNase-Free DNase Set, Qiagen). RNA concentrations were determined with a BioMate™ 3 spectrophotometer and analyzed by VISIONlite software (Thermo Fisher Scientific, Waltham, USA). 1 µg of total RNA of each sample was reverse transcribed into cDNA using Moloney Murine Leukemia Virus Reverse Transcriptase (Promega, Madison, USA) according to the manufacturer's protocol.

### Quantitative real-time polymerase chain reaction

Gene expression was analyzed by quantitative real-time polymerase chain reaction (qRT-PCR) using SYBR® Green-based RT^2^ qPCR Primer Assays (SABiosciences/Qiagen) for TLR1-10 and MyD88. Myxovirus-resistance protein (MX1) and hypoxanthine-guanine phosphoribosyltransferase (HPRT) served as positive control and housekeeping gene, respectively. qRT-PCR was performed using a 7500 Fast Real-Time PCR System (Applied Biosystems, Foster City, USA). PCR reactions were performed in duplicates, and results were expressed as the average of relative gene expression normalized for HPRT mRNA expression for each study subject and fold change of expression calculated by the 2^−ΔΔCt^ method [9]. Set-up experiments confirmed that mRNA expression levels of the housekeeping gene HPRT were not modulated by interferon-β treatment (data not shown).

### Flow cytometry

Cell surface phenotyping of PBMCs and intracellular expression of TLR7 and MyD88 protein was measured by flow cytometry. To detect pDCs (CD14−CD123+CD303+) and monocytes (CD14+), PBMCs were stained with anti-CD14 Pacific Blue antibody (Ab) (clone M5E5, BioLegend, San Diego, USA), anti-CD123 PerCP-Cy5.5 Ab (clone 6H6, eBioscience, San Diego, USA), and anti-CD303 APC Ab (clone AC144, Miltenyi Biotec, Bergisch Gladbach, Germany). To identify myeloid dendritic cells (mDCs, CD14−CD123^low^CD1c+CD11c^high^), PBMCs were stained with anti-CD14 Pacific Blue Ab, anti-CD123 PerCP-Cy5.5 Ab, anti-CD1c PE (clone L161, BioLegend), and anti-CD11c APC Ab (clone 3.9, BioLegend). To mark CD19+ B cells, CD4+, and CD8+ T cells, PBMCs were stained with anti-CD19 APC Ab (clone HIB19, eBioscience), anti-CD4 PerCP-Cy5.5 Ab (clone RPA-T4, BD Biosciences, Heidelberg, Germany), and anti-CD8 Pacific Blue Ab (clone RPA-T8, BD Biosciences). For intracellular staining cells were fixed, permeabilized using the BD Cytofix/Cytoperm™ Kit (BD Biosciences), and stained with anti-TLR7 Alexa488 Ab (clone 66H3, Dendritics, Lyon, France) or anti-MyD88 Alexa488 Ab (clone 4d6, Imgenex). An Alexa488-conjugated isotype (Imgenex) was used as a negative control.

Fc receptors were blocked with FcR Blocking Reagent (Miltenyi Biotec) before cell surface and intracellular staining. 5×10^5^ viable cells (healthy donor samples) and 3×10^5^ cells (patient samples) were acquired using a BD FACSCantoII cytometer (BD Biosciences, San Jose, USA) and analyzed using FlowJo9 software (Tree Star, Ashland, USA). All patient samples were coded and examiners were blinded to the patients' treatment status. Results were expressed as mean fluorescence intensity (MFI) with the level of expression calculated as delta (Δ) MFI = MFI^TLR7 or MyD88^ – MFI^isotype^.

### Analysis of cytokine production upon TLR7 stimulation

PBMCs (2×10^6^ cells/ml) were cultured for 12 hours with or without 1000 U/ml interferon-β, followed by stimulation with the TLR7 ligand loxoribine (0,1 mM, InVivoGen) for an additional 6 hours. Supernatants were harvested and stored at −80°C. Protein expression of interferon-α, tumor necrosis factor-α, interferon-γ-induced protein 10, macrophage inflammatory protein-1α, interleukin (IL) -6, IL-8, IL-10, IL-23, and IL-27 was determined in cell culture supernatants by using the multianalyte detection system FlowCytomix™ (eBioscience) according to the manufacturer's instructions. pDCs were analyzed for intracellular interferon-α production by flow cytometry staining with an anti-interferon-α FITC Ab (Miltenyi Biotec). Brefeldin A (5 µg/ml) was added to PBMCs for the final 3 hours of stimulation.

### Gene expression profiling with microarray analysis

Experimental details of the gene expression microarray analysis were previously described [10]. Briefly, 25 Caucasian individuals from a previously published cohort of patients treated with interferon-β-1b [10], [11] were included in this study (16 females, 9 males; mean age 39.6 years). They were diagnosed with RRMS according to the McDonald 2001 criteria [12] and started on a therapy with 250 µg interferon-β-1b administered subcutaneously every other day. 15 ml peripheral venous EDTA blood was obtained from each patient before and one month after initiation of therapy. The samples were always withdrawn prior to the interferon-β injection. PBMCs were isolated using Ficoll within one hour after blood withdrawal. Ficoll-isolated PBMCs were then lysed in RLT buffer (RNeasy kit) and stored at −80°C. Total RNA was later extracted from each sample using the RNeasy kit. Afterwards, samples of 7 µg total RNA were processed, labelled, and hybridized to Affymetrix HG-U133 A and B oligonucleotide arrays (Affymetrix, Santa Clara, USA). The arrays were scanned at 3 µm resolution with a Hewlett Packard GeneArray Scanner G2500A (Affymetrix).

### Microarray data pre-processing and analysis

For experimental details and published data regarding the affymetrix data array please see above (“Gene expression profiling with microarray analysis”). The raw Affymetrix microarray data were pre-processed by applying the MAS5.0 algorithm with a custom chip definition file (CDF). The custom CDF (http://www.xlab.unimo.it/GA_CDF/, version 2.1.0) was based on the information contained in the databases GeneAnnot (version 1.9) and GeneCards (version 2.41) [13]. Compared to the original CDF from Affymetrix, the custom CDF defines a set of probe sets, in which each probe set consists of all specific probes for one particular gene. This ensures a one-to-one correspondence between genes and custom probe sets. Data were normalized separately for the chip types A and B with a loess normalization using the package affy in the R software environment. Raw and pre-processed data are publicly available in MIAME format together with the patients' clinical data in the Gene Expression Omnibus (GEO) database (accession number GSE24427). Using these data we compared PBMC transcript levels of TLR1-10, MyD88, and MX1 after one month of interferon-β-1b treatment with pre-treatment levels.

### Statistics

For *in vitro* experiments significance of differences was assessed by paired *t*-test or one-way ANOVA with Tukey's post test. Differences of protein expression in untreated, interferon-β- or glatiramer acetate-treated patients were analyzed by Mann Whitney U test. *P*-values <0.05 were considered significant. Differences of gene expression determined by microarray before and during interferon-β therapy were analyzed by Wilcoxon signed-rank test at the α = 0.01 significance level.

## Results

### Interferon-β upregulates TLR3, TLR7, and MyD88 mRNA, but downregulates TLR6 and TLR9 mRNA in human mononuclear blood cells

**Figure 1:**
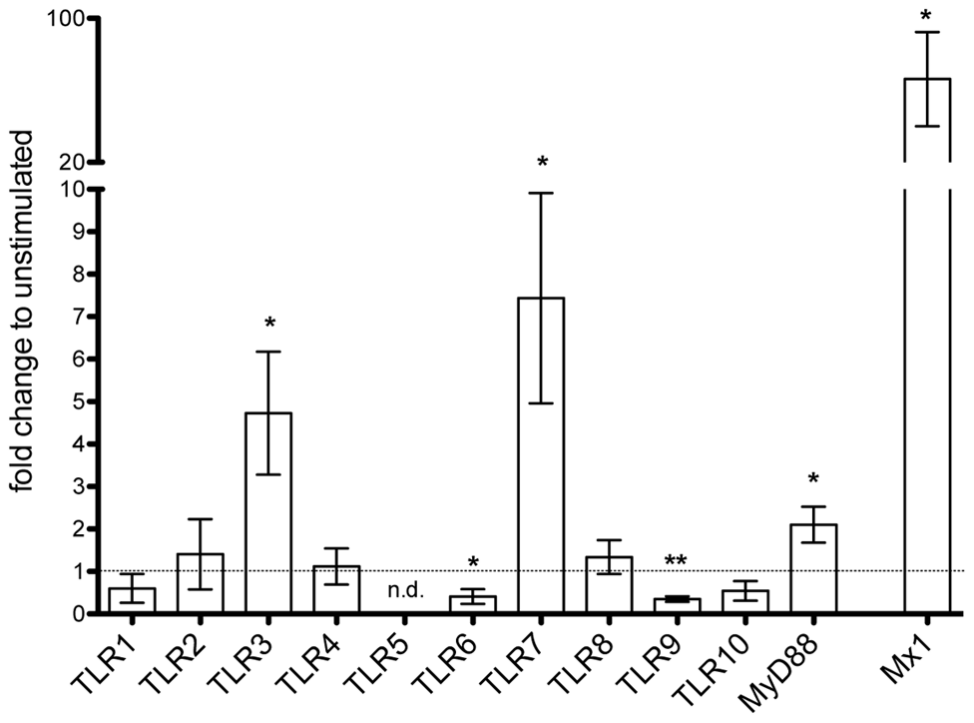
Differential regulation of TLR mRNA expression in human PBMCs by interferon-β. PBMCs from healthy donors were incubated with 1000 U/ml interferon-β. Untreated cells served as control. After 6 hours of treatment RNA was isolated and qRT-PCR performed with primers for TLR1-10, MyD88, and MX1. Results (mean±SD) from *n* = 3 independent experiments with PBMCs from different healthy donors are shown as fold-change to unstimulated controls. The dotted line marks the expression level in unstimulated control PBMCs, which was set to 1. Statistical significance of differences was assessed by paired *t*-test. *p<0.05; **p<0.01; n.d., not detectable.

To comprehensively analyze the effects of interferon-β on all known human TLRs (TLR1-10) and the TLR adaptor molecule MyD88, PBMCs from healthy donors were stimulated with 1000 U/ml interferon-β for 6 hours. Changes in mRNA expression levels were subsequently measured by qRT-PCR. The well-established interferon-β response gene MX1 [14] served as positive control and was strongly (66.2-fold) induced by interferon-β treatment, proving biological activity of the employed interferon-β preparation (Fig. 1). Compared to control conditions, interferon-β caused a significant increase of TLR3 (4.7-fold), TLR7 (7.4-fold), and MyD88 (2.1-fold) mRNA expression as well as a significant decrease of TLR6 (2.4-fold) and TLR9 (2.8-fold) mRNA expression in human PBMCs. While TLR5 mRNA was not detectable in human PBMCs, TLR1, TLR2, TLR4, TLR8, and TLR10 mRNA were expressed but not significantly regulated by interferon-β during the observed time period.

### Dose- and time-dependent modulation of TLR expression in human peripheral blood mononuclear cells by interferon-β

**Figure 2:**
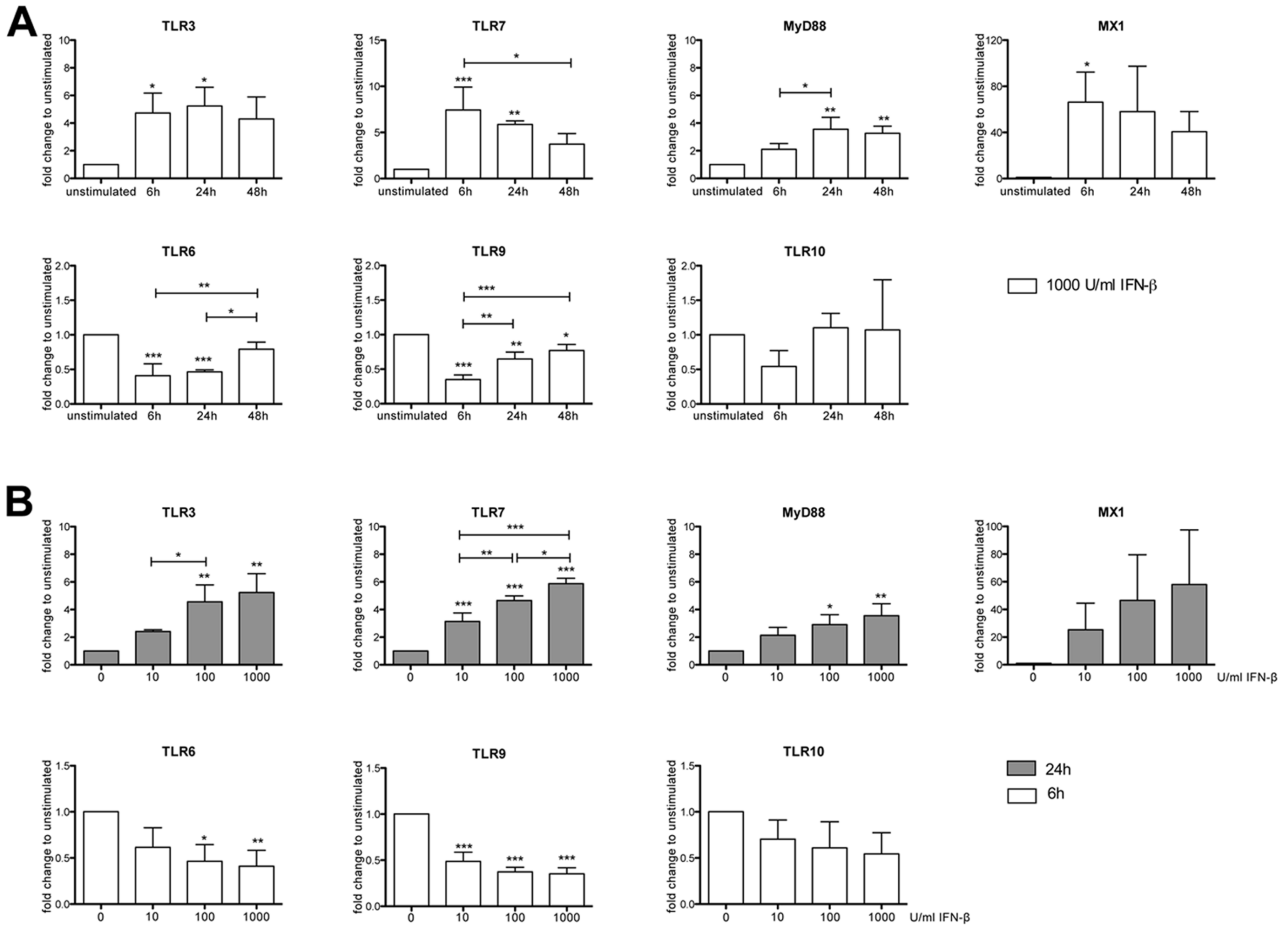
Dose- and time-dependent expression of TLR mRNA in PBMCs in response to interferon-β. (**A**) PBMCs from healthy donors were incubated with 1000 U/ml interferon-β. Control cells were left untreated. After 6, 24, and 48 hours RNA was isolated and qRT-PCR performed with primers for the indicated genes. (**B**) PBMCs were stimulated with 0, 10, 100, or 1000 U/ml interferon-β. After 6 hours (TLR6, TLR9, TLR10) or 24 hours (TLR3, TLR7, MyD88, MX1) RNA was isolated and qRT-PCR analysis performed. Results (mean±SD) from *n* = 3 independent experiments with PBMCs from different healthy donors are shown as fold-change to unstimulated controls. Statistical significance was assessed by one-way ANOVA with Tukey's post test. IFN-β, interferon-β; *p<0.05; **p<0.01; ***p<0.001.

To further characterize regulation of TLR expression by interferon-β a kinetic and dose-response study was performed for the targets significantly modulated by interferon-β as well as for TLR10. In PBMCs from healthy donors stimulated with 1000 U/ml interferon-β for 6, 24, and 48 hours the maximal response for TLR7 and MX1 mRNA was observed between 6 and 24 hours, while maximum mRNA levels of TLR3 and MyD88 were detectable after 6 and 24 hours as well as after 24 and 48 hours, respectively (Fig. 2A). The lowest expression levels of TLR6 and TLR9 mRNA were observed after 6 and 24 hours and after 6 hours, respectively. The regulatory effects of interferon-β were still detectable after 48 hours for MyD88 and TLR9 mRNA levels. Expression of TLR10 after 6, 24, and 48 hours of interferon-β stimulation was not significantly changed compared to unstimulated conditions. Treatment of PBMCs with various doses of interferon-β (0, 10, 100, or 1000 U/ml) resulted in a dose-response effect, with the strongest regulation observed at the highest concentration of interferon-β (Fig. 2B).

### Interferon-β induces TLR7 and MyD88 protein expression in plasmacytoid dendritic cells

**Figure 3:**
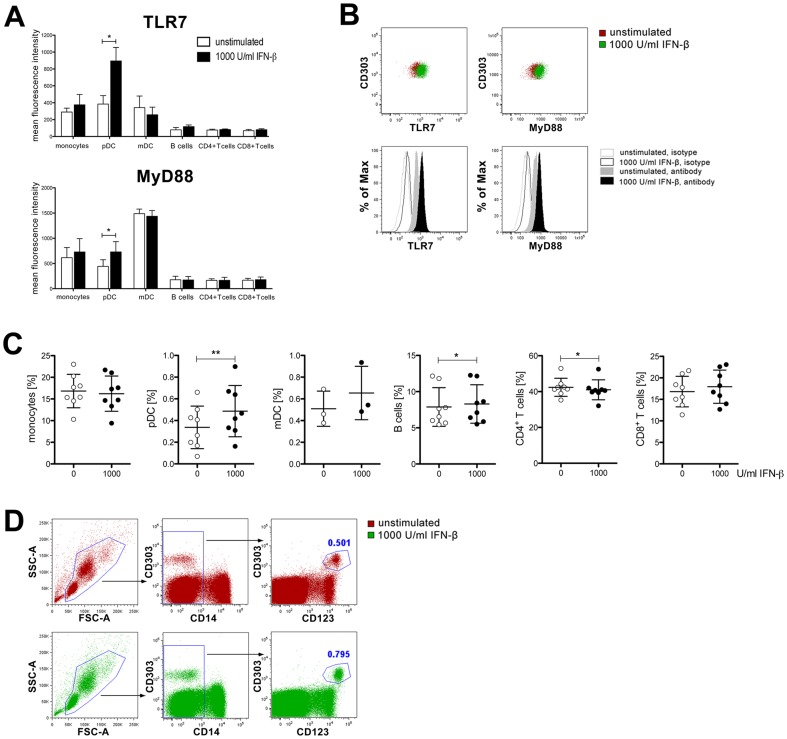
Interferon-β upregulates TLR7 and MyD88 protein expression in plasmacytoid dendritic cells *in vitro*. (**A, B, C, D**) PBMCs were either incubated with 1000 U/ml interferon-β or left untreated. (**A**) After 24 hours cells were stained with antibodies to TLR7 and MyD88 as well as isotype controls and analyzed by flow cytometry. Mean fluorescence intensity (MFI) was determined. Results are presented as mean±SD (MFI^TLR7 or MyD88^ – MFI^isotype^) from *n* = 3 healthy donors for each cell type and TLR. (**B**) Representative dot plots and histogram plots of TLR7 and MyD88 expression in pDCs. (**C**) Percentages of each cell population of all live cells are depicted with each dot representing one healthy donor. (**D**) Representative dot plots display the gating strategy for pDCs. Statistical significance was assessed by paired *t*-test. IFN-β, interferon-β; *p<0.05; **p<0.01.

Having established that interferon-β modulates TLR and MyD88 mRNA expression in PBMCs we were interested to know which leukocyte subsets are responsible for this effect and whether the observed changes in mRNA expression are translated into changes at the protein level. Therefore, we studied regulation of TLR7 and MyD88 expression by interferon-β in different leukocyte subsets by flow cytometry. Following incubation of PBMCs with 1000 U/ml interferon-β for 24 hours, a significant increase of TLR7 protein was observed specifically in pDCs (MFI ± SD unstimulated vs. 1000 U/ml interferon-β, 384.7±100.0 vs. 894.7±159.6), but not in the other leukocyte subsets (monocytes, mDCs, B cells, CD4+ T cells, CD8+ T cells) investigated (Fig. 3A, B). Likewise, treatment with interferon-β resulted in a significant upregulation of MyD88 (443.0±131.7 vs. 730.0±203.2) exclusively in pDCs. Different technical approaches such as direct analysis after stimulation or analysis after storage at −80°C for 7 days and subsequent thawing did not result in significantly different outcomes regarding TLR7 and MyD88 expression in pDCs in response to interferon-β (Fig. S1).

We also evaluated whether interferon-β treatment affects cell numbers of different leukocyte subsets *in vitro* by determining the percentage of each leukocyte subset among all live cells. Stimulation of PBMCs with interferon-β for 24 hours significantly increased the percentage of pDCs in the whole live cell population (Fig. 3C, D). Except for a slight but significant increase of B cells and a minor but significant decrease of CD4+ T cells no quantitative changes were observed in any other leukocyte subset.

### Regulation of TLR mRNA expression in interferon-β-treated patients with MS in vivo parallels the effects observed *in vitro*

**Figure 4:**
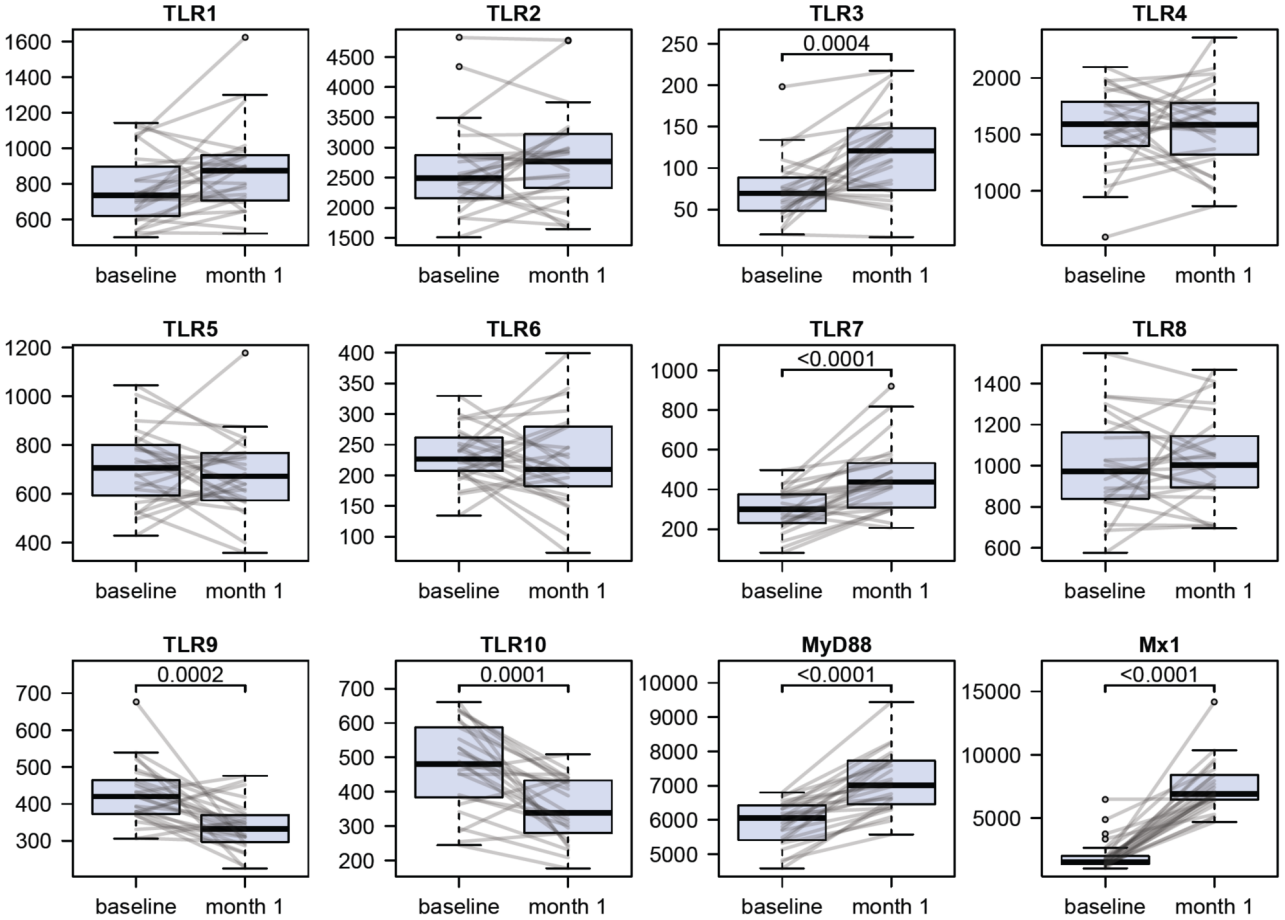
Regulation of TLR, MyD88, and MX1 mRNA expression in interferon-β-treated patients with MS. Expression levels of TLR1-10, MyD88, and MX1 mRNA were determined in PBMCs of 25 patients with RRMS by Affymetrix microarray analysis immediately before and one month after start of therapy with interferon-β-1b. Boxplots show medians, upper and lower quartiles as well as outliers, and graphically display the spread (interquartile range) of the pre-processed microarray data. Statistical significance of gene expression changes in response to therapy was assessed by Wilcoxon signed-rank test. *p*-values <0.01 are indicated.

To investigate whether regulation of TLRs *in vitro* is paralleled by similar changes in interferon-β-treated patients with MS we analyzed a data set from 25 patients with RRMS before and one month after start of treatment with interferon-β-1b [10], [11] for gene expression levels of TLR1-10, MyD88, and MX1. Interferon-β therapy was associated with a significant (*p*<0.01) upregulation of TLR3, TLR7, MyD88, and MX1 mRNA as well as a significant downregulation of TLR9 mRNA (Fig. 4). Furthermore, TLR10 mRNA was significantly downregulated in PBMCs of patients with RRMS after one month of therapy with interferon-β. In contrast to the observed downregulation of TLR6 mRNA by interferon-β in PBMCs *in vitro*, no influence of interferon-β on TLR6 expression was detected in patients with RRMS *in vivo*.

### Increased expression of TLR7 protein in plasmacytoid dendritic cells of interferon-β-treated patients with MS

**Figure 5:**
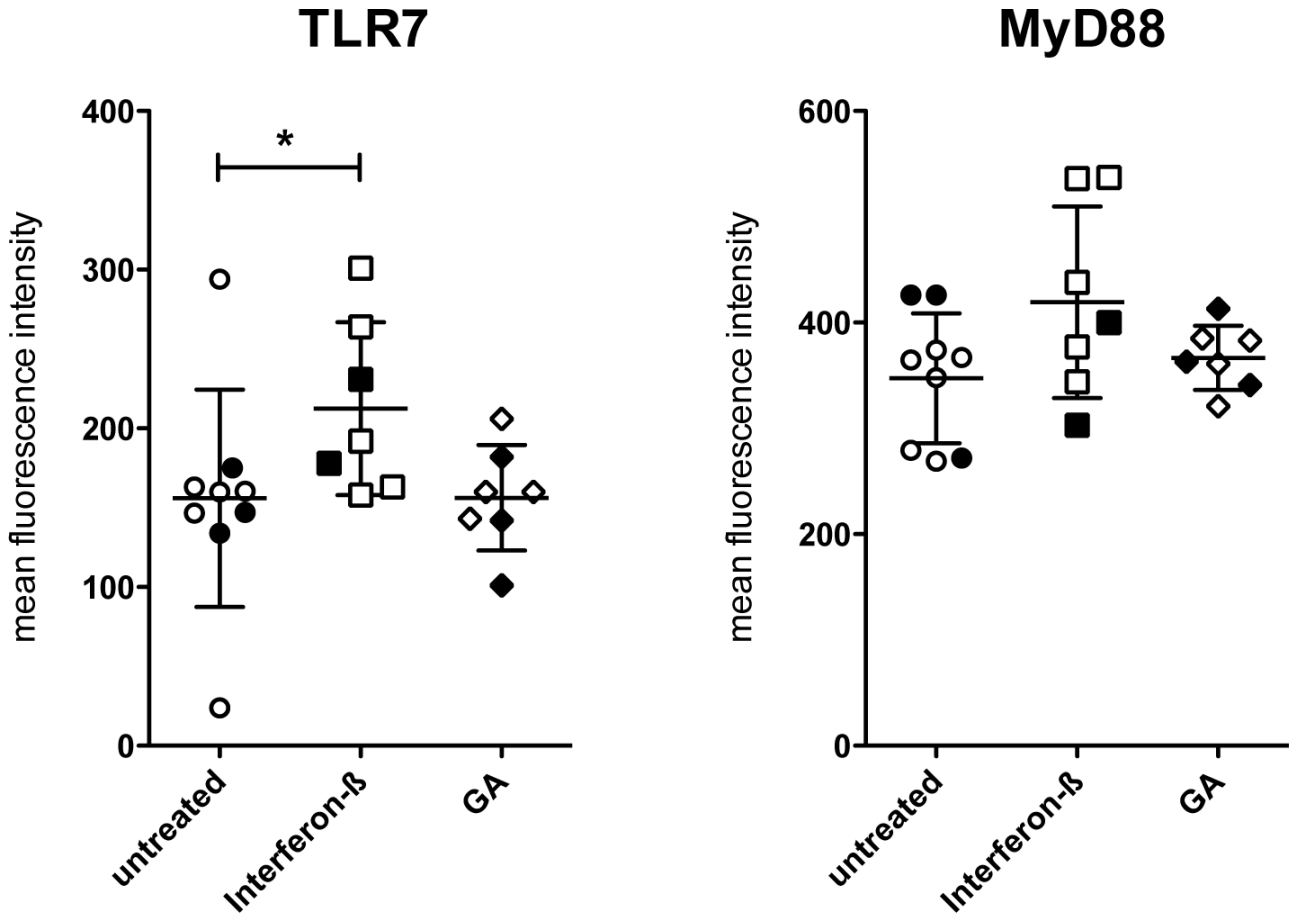
Increased TLR7 and MyD88 protein expression in plasmacytoid dendritic cells of interferon-β-treated patients with MS. Expression of TLR7 and MyD88 in pDCs of 9 untreated, 7 interferon-β-, and 7 glatiramer acetate-treated patients with CIS/RRMS was analyzed by flow cytometry. Results are expressed as MFI^TLR7 or MyD88^ – MFI^isotype^ with each dot representing one individual. Empty symbols represent female and filled symbols male individuals. Statistical significance of differences was assessed by Mann Whitney U test. GA, glatiramer acetate; *p<0.05.

We next investigated whether changes of interferon-β-induced mRNA expression in PBMCs of patients with MS may also be detectable at the protein level. Since the *in vitro* studies had demonstrated an upregulation of TLR7 and MyD88 protein expression by interferon-β in pDCs, we determined the expression levels of these proteins in pDCs of 9 untreated, 7 interferon-β-, and 7 glatiramer acetate-treated patients with a clinically isolated syndrome (CIS) or RRMS (see Table 1 for patient details) by flow cytometry. TLR7 expression was significantly (*p* = 0.039) higher in pDCs of patients treated with interferon-β than in untreated patients (Fig. 5). Elevated TLR7 expression in pDCs of interferon-β-treated as compared to glatiramer acetate-treated patients almost reached statistical significance (*p* = 0.053) as well. A trend for elevated expression levels under interferon-β treatment was also observed for MyD88 (*p* = 0.11, untreated vs. interferon-β; *p* = 0.32, interferon-β vs. glatiramer acetate). Nevertheless, it should be noted that the mean age in the glatiramer acetate-treated patient group was higher than in the other two groups (interferon-β, 30.1 years; glatiramer-acetate, 44.3 years; untreated, 37 years); thus, an impact of age cannot be ruled out at this point.

**Table 1:** Demographic and clinical details of patients shown in Figure 5.

Patients: Treatment (number of patients)	CIS/RRMS (number of patients)	Female/Male (number of patients)	Age (years) mean ± SD	EDSS median (range)	Type of interferon-β treatment (number of patients)
No treatment (9)	8/1	6/3	37±10.6	1 (0–2.5)	None
Glatiramer acetate (7)	3/4	4/3	44.3±10.4	2 (1–4)	None
Interferon-β (7)	5/2	5/2	30.1±5.6	1.5 (0–3)	Interferon-β-1a i.m. (2) Interferon-β-1a s.c. (3) Interferon-β-1b s.c. (2)

CIS, clinically isolated syndrome; RRMS, relapsing-remitting multiple sclerosis; EDSS, expanded disability status scale; i.m., intramuscular; s.c., subcutaneous.

### Pre-treatment with interferon-β leads to enhanced production of interferon-α upon TLR7 stimulation

**Figure 6:**
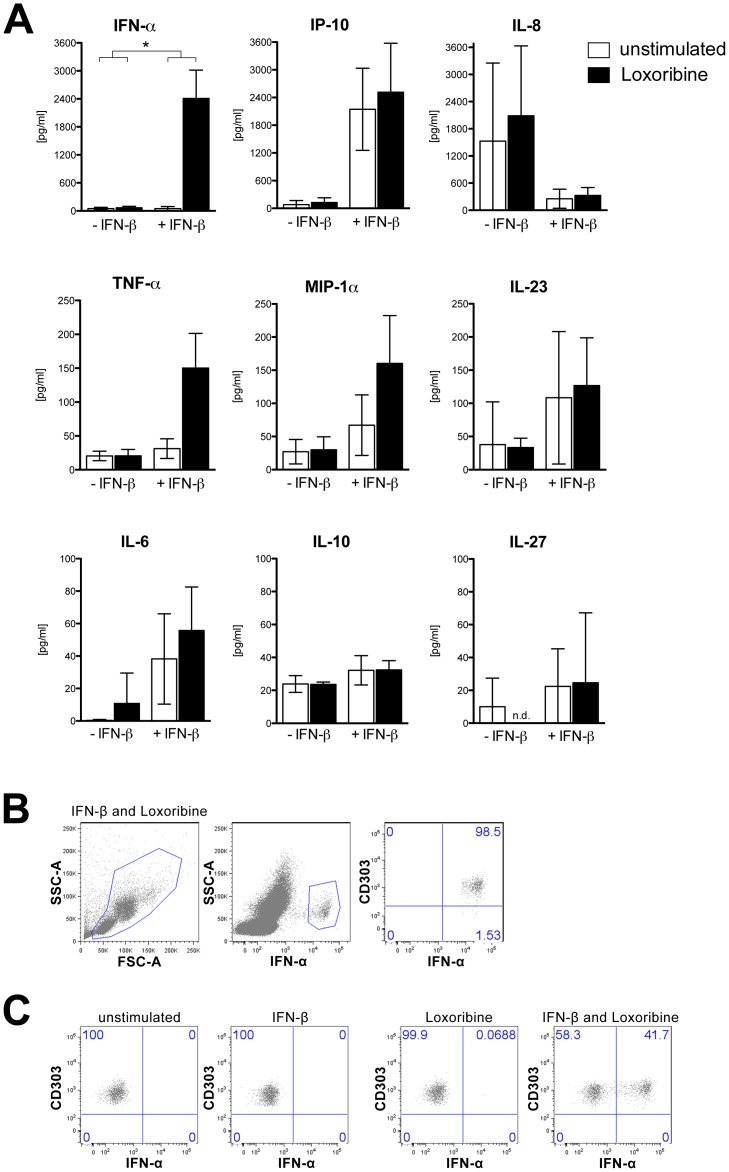
Interferon-β pre-treatment leads to strongly increased production of interferon-α upon TLR7 stimulation. (**A**) PBMCs from healthy donors were either incubated with 1000 U/ml interferon-β or left untreated for 12 hours. Subsequently, TLR7 was stimulated with 0,1 mM loxoribine for additional 6 hours. Amounts of cytokines and chemokines were determined in cell culture supernatants by bead-based immunoassays. Results shown are mean±SD from *n* = 3 healthy donors. For assessment of statistical significance, the values of loxoribine-untreated cells were subtracted from values of loxoribine-treated cells for both, cells with and without interferon-β pre-treatment, and the resulting differences compared by paired t-test. * *p*<0,05. (**B**, **C**) Cells were treated as in (**A**), but brefeldin A was added for the final 3 hours of stimulation before the intracellular expression of interferon-α was analyzed by flow cytometry. Representative dot plots showing (**B**) the percentage of CD303+ cells (pDCs) of all interferon-α positive cells and (**C**) the percentage of pDCs positive for interferon-α for one donor representative of *n* = 4. IFN-α, interferon-α; TNF-α, tumor necrosis factor-α; IFN-β, interferon-β; MIP-1α, macrophage inflammatory protein-1α, IP-10, interferon-γ-induced protein 10; IL, interleukin, n.d., not detected.

Finally, we investigated whether the observed upregulation of TLR7 in pDCs induced by interferon-β has a functional impact on the innate immune response, as measured by the production of cytokines (interferon-α, tumor necrosis factor-α, IL-6, IL-8, IL-10, IL-23, IL-27) and chemokines (interferon-γ-induced protein 10 and macrophage inflammatory protein-1α). To this end, we induced upregulation of TLR7 by incubating PBMCs of healthy donors with interferon-β and subsequently stimulated these cells with the TLR7-specific ligand loxoribine. Analysis by bead-based immunoassays revealed that the production of interferon-α was strongly increased in cells pre-treated with interferon-β and subsequently stimulated with loxoribine (*p* = 0.024; Fig. 6A). A similar, almost significant (*p* = 0.06), effect was also observed for tumor necrosis factor-α. In addition, macrophage inflammatory protein-1α showed a trend for upregulation in loxoribine-treated cells prestimulated with interferon-β (*p* = 0.11). Expression of the other cytokines and chemokines analyzed did not appear to be enhanced by successive stimulation with these two agents. Treatment with interferon-β or loxoribine alone had no effects on the release of interferon-α and tumor necrosis factor-α. Using intracellular flow cytometry, pDCs were identified as the almost exclusive cellular source of the enhanced release of interferon-α in response to the combined treatment with interferon-β and loxoribine (Fig. 6B, C).

## Discussion

Since interferon-β has genuine antiviral properties [15] and TLRs play a prominent role in antiviral immunity [6] we hypothesized that interferon-β may be involved in the regulation of TLRs, which could be one mechanism underlying the beneficial effects of interferon-β in MS. Indeed, we herein show that interferon-β specifically and differentially modulates TLR mRNA expression in PBMCs of healthy donors and has almost identical effects on the expression of TLR mRNA in PBMCs of interferon-β-treated patients with RRMS. In keeping with its antiviral properties, the TLRs most strongly and consistently regulated by interferon-β (TLR3, TLR7, and TLR9) contribute to innate immunity against viruses. Located on the membrane of intracellular vesicles, in particular endolysosomes, these TLRs recognize viral double-stranded RNA (TLR3), viral single-stranded RNA (TLR7), or virus- or bacteria-derived DNA (TLR9) [6]. It therefore seems likely that the observed changes of TLR3, TLR7, TLR9, and MyD88 mRNA expression are part of the antiviral defence program induced by interferon-β.

Flow cytometric studies on the effects of interferon-β on TLR protein expression and on the leukocyte subsets primarily responding to interferon-β demonstrated that interferon-β upregulates TLR7 and MyD88 protein specifically in pDCs. Again, these *in vitro* observations correlated with results obtained in interferon-β-treated patients with MS whose pDCs displayed a higher expression level of TLR7 than pDCs of untreated patients. Since patients treated with glatiramer acetate, an alternative MS therapeutic, did not exhibit a comparable increase of TLR7 in pDCs, this effect appears to be specific for interferon-β. While it is known that interferon-α upregulates TLR7 and MyD88 expression in naive B cells [16] and that interferon-β increases the expression of functionally active TLR7 and MyD88 in human mDCs [17], [18], upregulation of TLR7 and MyD88 protein in pDCs by interferon-β in pDCs of healthy donors *in vitro* and increased levels of TLR7 in pDCs of interferon-β-treated patients *in vivo* is a novel finding that, to the best of our knowledge, has not been reported before. Of note, since whole populations of PBMCs were stimulated in our experiments, we cannot discard the possibility that the interferon-β-induced upregulation of TLR7 and MyD88 in pDCs might be indirect. Further studies with purified PBMC subsets will be required to analyse the involvement and exact function of distinct cell populations in this context. Also, the immune cell populations responsible for regulation of TLR3 and TLR9 expression in response to interferon-β in our experiments were not identified. TLR9 is known to be expressed in pDCs and B cells, and TLR3 expression was observed in NK and T cells as well as in DCs [21]–[26]. Therefore, these cells might up- or downregulate the respective receptors in response to interferon-β. Finally, animal models of MS may aid in the elucidation of functional aspects of the interaction of interferon-β and TLRs [19], [20].

Although pDCs account for less than 1% of all circulating mononuclear blood cells (see Fig. 3C), they represent the principal PBMC subset involved in antiviral innate immunity and are capable of releasing large amounts of type I interferon in response to TLR activation [27], [28]. We therefore investigated whether upregulation of TLR7 in pDCs by interferon-β may further sensitize these cells to the activation of this receptor by its specific ligands. Indeed, subsequent stimulation of interferon-β-treated PBMCs with the TLR7 agonist loxoribine resulted in a strongly enhanced production of interferon-α as well as a less pronounced increase of tumor necrosis factor-α. Flow cytometry unambiguously confirmed that pDCs were the main cell type responsible for the increased release of interferon-α following TLR7 activation after interferon-β pre-treatment. The fact that loxoribine treatment alone did not induce the release of interferon-α from PBMCs suggests that interferon-β may serve as an important co-factor for TLR7 activation in human pDCs, which is similar to the interferon-β-induced sensitization to the TLR7 agonist 3M-001 previously described in human mDCs [18]. Production of interferon-α by human dendritic cells upon TLR7 ligation therefore appears to be tightly controlled and to depend on a further signal, such as interferon-β, in addition to TLR7 stimulation. Altogether, our findings strongly suggest that upregulation of TLR7 by interferon-β is functionally relevant, as it is associated with an increased sensitivity towards TLR7 activation, thereby leading to an increased production of specific cytokines which may result in an amplified innate immune response. Interestingly, pDCs were reported to accumulate in white matter lesions and leptomeninges of patients with MS and numbers of pDCs were increased in the cerebrospinal fluid of untreated MS patients during relapse [29], [30], indicating that pDCs could on the one hand play a role in MS and on the other could thus represent a relevant target for interferon-β therapy.

The question arises how these findings may be related to the beneficial effects, i.e. relapse reduction, by interferon-β in patients with MS. There is strong and consistent evidence for an increased risk of MS relapses around the time of viral infections [31], [32]. It therefore seems conceivable that upregulation of TLR7 in pDCs by interferon-β and a subsequently enhanced antiviral innate immune response may reduce the risk of virus-triggered MS relapses. As one example in accordance with this hypothesis, there is a temporal relationship between infections with influenza A virus and MS relapses [33], and single-stranded RNA derived from influenza A virus is a known TLR7 ligand [34]. One could therefore speculate that an enhanced immune response against influenza A virus in patients with MS treated with interferon-β could reduce the risk of influenza A virus-triggered relapses. In accordance with the concept of an interferon-β-induced enhanced antiviral state several further genes involved in antiviral detection and defence mechanisms such as OAS1-3, GBP1, IFIT1, DDX58, IRF7 and RSAD2 were found to be upregulated after interferon-β treatment in a former analysis of our array data set [10], [35]–[39].

It was previously shown that interferon-β inhibits TLR9 processing in pDCs, resulting in decreased activation of pDCs by a TLR9 agonist [40]. The authors of this work proposed that reduced activation of pDCs by viral pathogens in interferon-β-treated patients with MS might thus affect the frequency of MS exacerbations. However, our present data indicate that the regulation of TLRs by interferon-β involves differential effects on distinct TLRs including TLR3, TLR7, TLR9, and possibly TLR10. Furthermore, our results indicate that interferon-β-induced upregulation of TLR7 is associated with enhanced activation of pDCs by a TLR7 agonist. The functional consequences of the interferon-β-induced regulation of different TLRs may thus differ and have to be taken into account for each TLR separately.

Increasing evidence suggests that TLRs may also be activated by host-derived molecules, such as endogenous nucleic acids bound by autoantibodies, and this phenomenon is thought to play a crucial role in the development and maintenance of autoimmune diseases [7]. We have recently demonstrated that endogenous single-stranded RNA such as miRNA is capable of activating TLR7 in the CNS, thereby inducing inflammation and neuronal injury [41]. It is currently unknown whether stimulation of TLRs by endogenous molecules plays a role in MS, but if this should be the case, modulation of TLRs by interferon-β may obviously influence such interactions.

In summary, our work, including both studies on immune cells from healthy donors and on patients with MS, implicates that modulation of the innate immune response may be a relevant mechanism of action of interferon-β in patients with MS and suggests a role for the innate immune receptor TLR7 as an important target in this context. We hypothesize that an enhanced antiviral immune response associated with upregulation of TLR7 in pDCs by interferon-β may reduce the frequency of virus-triggered MS relapses. Further studies on the functional consequences of the specific and differential regulation of TLRs by interferon-β may not only shed more light on the regulation of the innate immune response by interferon-β, but possibly also on pathogenic mechanisms operating in MS.

## Supporting Information

Figure S1 **Different technical approaches such as direct analysis after stimulation or analysis after freezing/thawing do not result in a different outcome regarding TLR7 and MyD88 expression in pDCs in response to interferon-β.** PBMCs from healthy donors were either incubated with 1000 U/ml interferon-β or left untreated. After 24 hours cells were either directly analyzed by flow cytometry or were frozen as described in the *Material and Methods* section, stored for 7 days at −80°C, and subsequently thawed and analyzed by flow cytometry. For flow cytometry PBMCs were stained with antibodies to identify pDCs, TLR7, and MyD88 as well as isotype controls. Mean fluorescence intensity (MFI) was determined and fold change of MFI in interferon-β-treated to untreated was calculated. Results are presented as mean±SD from *n* = 3 healthy donors for pDCs. Analysis by paired *t*-tests showed no significant differences between directly analyzed and frozen/thawed PBMC.
(DOC)

## References

[1] Isaacs A, Lindenmann J (1957) Virus interference. I. The interferon. Proc R Soc Lond B Biol Sci 147: 258–267. 26297790

[2] Jacobs LD, Cookfair DL, Rudick RA, Herndon RM, Richert JR, et al (1996) Intramuscular interferon beta-1a for disease progression in relapsing multiple sclerosis. Ann Neurol 39: 285–294. 8602746 10.1002/ana.410390304

[3] The IFNB Multiple Sclerosis Study Group (1993) Interferon beta-1b is effective in relapsing-remitting multiple sclerosis. I. Clinical results of a multicenter, randomized, double-blind, placebo-controlled trial. Neurology 43: 655–661. 8469318 10.1212/wnl.43.4.655

[4] PRISMS (Prevention of Relapses and Disability by Interferon beta-1a Subcutaneously in Multiple Sclerosis) Study Group (1998) Randomised double-blind placebo-controlled study of interferon beta-1a in relapsing/remitting multiple sclerosis. Lancet 352: 1498–1504. 9820297

[5] Rudick RA, Goelz SE (2011) Beta-interferon for multiple sclerosis. Exp Cell Res 317: 1301–1311. 21396360 10.1016/j.yexcr.2011.03.002

[6] Kawai T, Akira S (2010) The role of pattern-recognition receptors in innate immunity: update on Toll-like receptors. Nat Immunol 11: 373–384. 20404851 10.1038/ni.1863

[7] Takeuchi O, Akira S (2010) Pattern recognition receptors and inflammation. Cell 140: 805–820. 20303872 10.1016/j.cell.2010.01.022

[8] Polman CH, Reingold SC, Edan G, Filippi M, Hartung HP, et al (2005) Diagnostic criteria for multiple sclerosis: 2005 revisions to the “McDonald Criteria”. Ann Neurol 58: 840–846. 16283615 10.1002/ana.20703

[9] Livak KJ, Schmittgen TD (2001) Analysis of relative gene expression data using real-time quantitative PCR and the 2(−Delta Delta C(T)) Method. Methods 25: 402–408. 11846609 10.1006/meth.2001.1262

[10] Goertsches RH, Hecker M, Koczan D, Serrano-Fernandez P, Moeller S, et al (2010) Long-term genome-wide blood RNA expression profiles yield novel molecular response candidates for IFN-beta-1b treatment in relapsing remitting MS. Pharmacogenomics 11: 147–161. 20136355 10.2217/pgs.09.152

[11] Hundeshagen A, Hecker M, Paap BK, Angerstein C, Kandulski O, et al (2012) Elevated type I interferon-like activity in a subset of multiple sclerosis patients: molecular basis and clinical relevance. J Neuroinflammation 9: 140. 22727118 10.1186/1742-2094-9-140 PMC3464734

[12] McDonald WI, Compston A, Edan G, Goodkin D, Hartung HP, et al (2001) Recommended diagnostic criteria for multiple sclerosis: guidelines from the international panel on the diagnosis of multiple sclerosis. Ann Neurol 50: 121–127. 11456302 10.1002/ana.1032

[13] Ferrari F, Bortoluzzi S, Coppe A, Sirota A, Safran M, et al (2007) Novel definition files for human GeneChips based on GeneAnnot. BMC Bioinformatics 8: 446. 18005434 10.1186/1471-2105-8-446 PMC2216044

[14] Haller O, Kochs G (2002) Interferon-induced mx proteins: dynamin-like GTPases with antiviral activity. Traffic 3: 710–717. 12230469 10.1034/j.1600-0854.2002.31003.x

[15] Haller O, Kochs G, Weber F (2006) The interferon response circuit: induction and suppression by pathogenic viruses. Virology 344: 119–130. 16364743 10.1016/j.virol.2005.09.024 PMC7125643

[16] Bekeredjian-Ding IB, Wagner M, Hornung V, Giese T, Schnurr M, et al (2005) Plasmacytoid dendritic cells control TLR7 sensitivity of naive B cells via type I IFN. J Immunol 174: 4043–4050. 15778362 10.4049/jimmunol.174.7.4043

[17] Zhang X, Jin J, Tang Y, Speer D, Sujkowska D, et al (2009) IFN-beta1a inhibits the secretion of Th17-polarizing cytokines in human dendritic cells via TLR7 up-regulation. J Immunol 182: 3928–3936. 19265172 10.4049/jimmunol.0802226

[18] Severa M, Remoli ME, Giacomini E, Annibali V, Gafa V, et al (2007) Sensitization to TLR7 agonist in IFN-beta-preactivated dendritic cells. J Immunol 178: 6208–6216. 17475848 10.4049/jimmunol.178.10.6208

[19] O'Brien K, Fitzgerald D, Rostami A, Gran B (2010) The TLR7 agonist, imiquimod, increases IFN-beta production and reduces the severity of experimental autoimmune encephalomyelitis. J Neuroimmunol 221: 107–111. 20138374 10.1016/j.jneuroim.2010.01.006

[20] Hayashi T, Yao S, Crain B, Chan M, Tawatao RI, et al (2012) Treatment of autoimmune inflammation by a TLR7 ligand regulating the innate immune system. PLoS One 7: e45860. 23029281 10.1371/journal.pone.0045860 PMC3461028

[21] Jarrossay D, Napolitani G, Colonna M, Sallusto F, Lanzavecchia A (2001) Specialization and complementarity in microbial molecule recognition by human myeloid and plasmacytoid dendritic cells. Eur J Immunol 31: 3388–3393. 11745357 10.1002/1521-4141(200111)31:11<3388::aid-immu3388>3.0.co;2-q

[22] Krug A, Towarowski A, Britsch S, Rothenfusser S, Hornung V, et al (2001) Toll-like receptor expression reveals CpG DNA as a unique microbial stimulus for plasmacytoid dendritic cells which synergizes with CD40 ligand to induce high amounts of IL-12. Eur J Immunol 31: 3026–3037. 11592079 10.1002/1521-4141(2001010)31:10<3026::aid-immu3026>3.0.co;2-h

[23] Hornung V, Rothenfusser S, Britsch S, Krug A, Jahrsdorfer B, et al (2002) Quantitative expression of toll-like receptor 1–10 mRNA in cellular subsets of human peripheral blood mononuclear cells and sensitivity to CpG oligodeoxynucleotides. J Immunol 168: 4531–4537. 11970999 10.4049/jimmunol.168.9.4531

[24] Muzio M, Bosisio D, Polentarutti N, D'Amico G, Stoppacciaro A, et al (2000) Differential expression and regulation of toll-like receptors (TLR) in human leukocytes: selective expression of TLR3 in dendritic cells. J Immunol 164: 5998–6004. 10820283 10.4049/jimmunol.164.11.5998

[25] Zarember KA, Godowski PJ (2002) Tissue expression of human Toll-like receptors and differential regulation of Toll-like receptor mRNAs in leukocytes in response to microbes, their products, and cytokines. J Immunol 168: 554–561. 11777946 10.4049/jimmunol.168.2.554

[26] Jongbloed SL, Kassianos AJ, McDonald KJ, Clark GJ, Ju X, et al (2010) Human CD141+ (BDCA-3)+ dendritic cells (DCs) represent a unique myeloid DC subset that cross-presents necrotic cell antigens. J Exp Med 207: 1247–1260. 20479116 10.1084/jem.20092140 PMC2882828

[27] Barchet W, Cella M, Colonna M (2005) Plasmacytoid dendritic cells-virus experts of innate immunity. Semin Immunol 17: 253–261. 15990333 10.1016/j.smim.2005.05.008

[28] Liu YJ (2005) IPC: professional type 1 interferon-producing cells and plasmacytoid dendritic cell precursors. Annu Rev Immunol 23: 275–306. 15771572 10.1146/annurev.immunol.23.021704.115633

[29] Lande R, Gafa V, Serafini B, Giacomini E, Visconti A, et al (2008) Plasmacytoid dendritic cells in multiple sclerosis: intracerebral recruitment and impaired maturation in response to interferon-beta. J Neuropathol Exp Neurol 67: 388–401. 18431257 10.1097/NEN.0b013e31816fc975

[30] Longhini AL, von Glehn F, Brandao CO, de Paula RF, Pradella F, et al (2011) Plasmacytoid dendritic cells are increased in cerebrospinal fluid of untreated patients during multiple sclerosis relapse. J Neuroinflammation 8: 2. 21214939 10.1186/1742-2094-8-2 PMC3022734

[31] Sibley A, Bamford CR, Clark K (1985) Clinical viral infections and multiple sclerosis. The Lancet 1 8441: 1313–1315. 10.1016/S0140-6736(85)92801-6 PMC7173199 2860501

[32] Rutschmann OT, McCrory DC, Matchar DB (2002) Immunization and MS: a summary of published evidence and recommendations. Neurology 59: 1837–1843. 12499473 10.1212/wnl.59.12.1837

[33] Oikonen M, Laaksonen M, Aalto V, Ilonen J, Salonen R, et al (2011) Temporal relationship between environmental influenza A and Epstein-Barr viral infections and high multiple sclerosis relapse occurrence. Mult Scler 17: 672–680. 21212088 10.1177/1352458510394397

[34] Lund JM, Alexopoulou L, Sato A, Karow M, Adams NC, et al (2004) Recognition of single-stranded RNA viruses by Toll-like receptor 7. Proc Natl Acad Sci U S A 101: 5598–5603. 15034168 10.1073/pnas.0400937101 PMC397437

[35] Sadler AJ, Williams BR (2008) Interferon-inducible antiviral effectors. Nat Rev Immunol 8: 559–568. 18575461 10.1038/nri2314 PMC2522268

[36] Itsui Y, Sakamoto N, Kakinuma S, Nakagawa M, Sekine-Osajima Y, et al (2009) Antiviral effects of the interferon-induced protein guanylate binding protein 1 and its interaction with the hepatitis C virus NS5B protein. Hepatology 50: 1727–1737. 19821486 10.1002/hep.23195

[37] Pichlmair A, Lassnig C, Eberle CA, Gorna MW, Baumann CL, et al (2011) IFIT1 is an antiviral protein that recognizes 5′-triphosphate RNA. Nat Immunol 12: 624–630. 21642987 10.1038/ni.2048

[38] Loo YM, Gale M Jr (2011) Immune signaling by RIG-I-like receptors. Immunity 34: 680–692. 21616437 10.1016/j.immuni.2011.05.003 PMC3177755

[39] Chin KC, Cresswell P (2001) Viperin (cig5), an IFN-inducible antiviral protein directly induced by human cytomegalovirus. Proc Natl Acad Sci U S A 98: 15125–15130. 11752458 10.1073/pnas.011593298 PMC64994

[40] Balashov KE, Aung LL, Vaknin-Dembinsky A, Dhib-Jalbut S, Weiner HL (2010) Interferon-beta inhibits toll-like receptor 9 processing in multiple sclerosis. Ann Neurol 68: 899–906. 21061396 10.1002/ana.22136 PMC3058378

[41] Lehmann SM, Kruger C, Park B, Derkow K, Rosenberger K, et al (2012) An unconventional role for miRNA: let-7 activates Toll-like receptor 7 and causes neurodegeneration. Nat Neurosci 15: 827–835. 22610069 10.1038/nn.3113

