# Supplementary material for: Multiple Sclerosis: Modulation of Toll-Like Receptor (TLR) Expression by Interferon-β Includes Upregulation of TLR7 in Plasmacytoid Dendritic Cells
Source: PLoS One. 2013 Aug 12;8(8):e70626. doi: 10.1371/journal.pone.0070626 (PMC3741212; doi:10.1371/journal.pone.0070626)
Supplement: Figure S1 — Different technical approaches such as direct analysis after stimulation or analysis after freezing/thawing do not result in a different outcome regarding TLR7 and MyD88 expression in pDCs in response to interferon-β. PBMCs from healthy donors were either incubated with 1000 U/ml interferon-β or left untreated. After 24 hours cells were either directly analyzed by flow cytometry or were frozen as described in the Material and Methods section, stored for 7 days at −80°C, and subsequently thawed and analyzed by flow cytometry. For flow cytometry PBMCs were stained with antibodies to identify pDCs, TLR7, and MyD88 as well as isotype controls. Mean fluorescence intensity (MFI) was determined and fold change of MFI in interferon-β-treated to untreated was calculated. Results are presented as mean±SD from n = 3 healthy donors for pDCs. Analysis by paired t-tests showed no significant differences between directly analyzed and frozen/thawed PBMC. (DOC) [file pone.0070626.s001.doc]

**Figure S1**


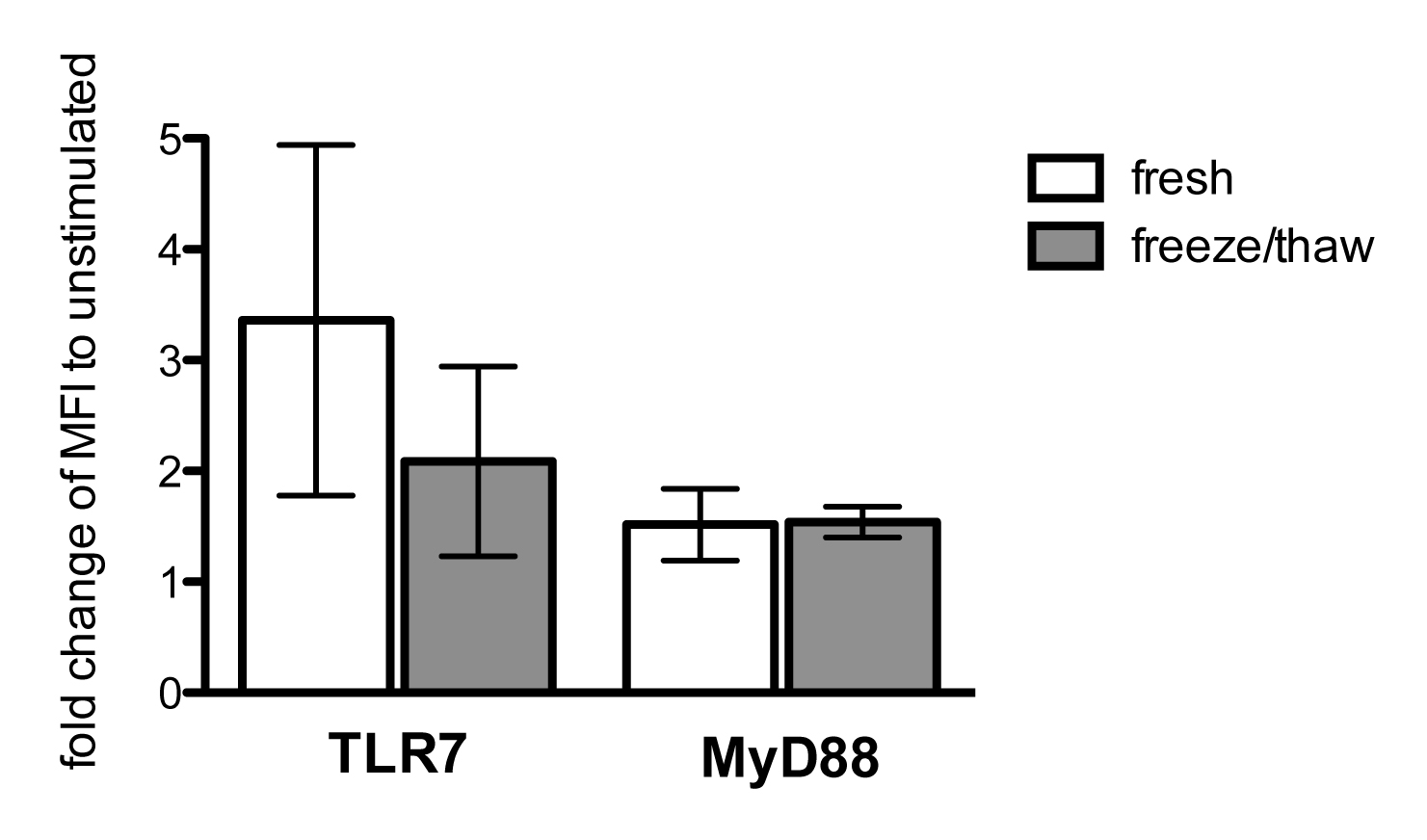


**Different technical approaches such as direct analysis after stimulation or analysis after freezing/thawing do not result in a different outcome regarding TLR7 and MyD88 expression in pDCs in response to interferon-β.**

PBMCs from healthy donors were either incubated with 1000 U/ml interferon-β or left untreated. After 24 hours cells were either directly analyzed by flow cytometry or were frozen as described in the *Material and Methods* section, stored for 7 days at -80°C, and subsequently thawed and analyzed by flow cytometry. For flow cytometry PBMCs were stained with antibodies to identify pDCs, TLR7, and MyD88 as well as isotype controls. Mean fluorescence intensity (MFI) was determined and fold change of MFI in interferon-β-treated to untreated was calculated. Results are presented as mean±SD from *n*=3 healthy donors for pDCs. Analysis by paired *t*-tests showed no significant differences between directly analyzed and frozen/thawed PBMC.
